# Diet quality and cardiovascular disease risk among breast cancer survivors in the Pathways Study

**DOI:** 10.1093/jncics/pkae013

**Published:** 2024-04-17

**Authors:** Isaac J Ergas, Richard K Cheng, Janise M Roh, Lawrence H Kushi, Jacob K Kresovich, Carlos Iribarren, Mai Nguyen-Huynh, Jamal S Rana, Eileen Rillamas-Sun, Cecile A Laurent, Valerie S Lee, Charles P Quesenberry, Heather Greenlee, Marilyn L Kwan

**Affiliations:** Division of Research, Kaiser Permanente Northern California, Oakland, CA, USA; University of Washington Medical Center, Seattle, WA, USA; Division of Research, Kaiser Permanente Northern California, Oakland, CA, USA; Division of Research, Kaiser Permanente Northern California, Oakland, CA, USA; H. Lee Moffitt Cancer Center and Research Institute, Tampa, FL, USA; Division of Research, Kaiser Permanente Northern California, Oakland, CA, USA; Division of Research, Kaiser Permanente Northern California, Oakland, CA, USA; Division of Research, Kaiser Permanente Northern California, Oakland, CA, USA; Oakland Medical Center, Oakland, CA, USA; Division of Public Health Sciences, Fred Hutchinson Cancer Center, Seattle, WA, USA; Division of Research, Kaiser Permanente Northern California, Oakland, CA, USA; Division of Research, Kaiser Permanente Northern California, Oakland, CA, USA; Division of Research, Kaiser Permanente Northern California, Oakland, CA, USA; University of Washington Medical Center, Seattle, WA, USA; Division of Public Health Sciences, Fred Hutchinson Cancer Center, Seattle, WA, USA; Division of Research, Kaiser Permanente Northern California, Oakland, CA, USA

## Abstract

**Background:**

Women with breast cancer are at higher risk of cardiovascular disease (CVD) compared with women without breast cancer. Whether higher diet quality at breast cancer diagnosis lowers this risk remains unknown. We set out to determine if higher diet quality at breast cancer diagnosis was related to lower risk of CVD and CVD-related death.

**Methods:**

This analysis included 3415 participants from the Pathway Study, a prospective cohort of women diagnosed with invasive breast cancer at Kaiser Permanente Northern California between 2005 and 2013 and followed through December 31, 2021. Scores from 5 diet quality indices consistent with healthy eating were obtained at the time of breast cancer diagnosis. Scores were categorized into ascending quartiles of concordance for each diet quality index, and multivariable adjusted hazard ratios (HRs) and 95% confidence intervals (CIs) were estimated. *P* values were 2-sided.

**Results:**

The Dietary Approaches to Stop Hypertension diet quality index was associated with lower risk of heart failure (HR = 0.53, 95% CI = 0.33 to 0.87; *P*_trend_ = .03), arrhythmia (HR = 0.77, 95% CI = 0.62 to 0.94; *P*_trend_ = .008), cardiac arrest (HR = 0.77, 95% CI = 0.61 to 0.96; *P*_trend_ = .02), valvular heart disease (HR = 0.79, 95% CI = 0.64 to 0.98; *P*_trend_ = .046), venous thromboembolic disease (HR = 0.75, 95% CI = 0.60 to 0.93; *P*_trend_ = .01), and CVD-related death (HR = 0.70, 95% CI = 0.50 to 0.99; *P*_trend_ = .04), when comparing the highest with lowest quartiles. Inverse associations were also found between the healthy plant-based dietary index and heart failure (HR = 0.60, 95% CI = 0.39 to 0.94; *P*_trend_* *=* *.02), as well as the alternate Mediterranean dietary index and arrhythmia (HR = 0.74, 95% CI = 0.60 to 0.93; *P*_trend_* *=* *.02).

**Conclusion:**

Among newly diagnosed breast cancer patients, higher diet quality at diagnosis was associated with lower risk of CVD events and death.

Currently, more than 3.8 million female breast cancer survivors live in the United States, and nearly 300 000 new breast cancer cases are expected in 2023 (1). Cardiovascular disease (CVD) is a major health concern for women who have survived their breast cancer, as it is the number one cause of non-breast cancer–related death in this population (2). Also, women with breast cancer are at higher risk of CVD compared with women without breast cancer (3,4), which may be due to the cardiotoxic effects of breast cancer treatment, as well as common risk factors shared by breast cancer and CVD (3-6).

Dietary guidance for women surviving breast cancer is limited and, until recently, was based primarily on research related to general cancer prevention (7). Over the past decade, a growing body of research has shown that breast cancer survivors with healthier dietary patterns tend to be at lower risk of non-breast cancer and overall mortality (8-12). Still, a major research gap remains regarding the relationship between diet quality and CVD-specific outcomes among women with breast cancer.

One approach for assessing the healthfulness of one’s diet, or diet quality, is calculating indices that characterize concordance with healthful eating recommendations (13). These indices are calculated from the quantities, portions, and frequencies of one’s dietary intakes (13) and are comprehensive measures of overall diet quality capturing unique information that extends beyond individual food items and nutrients.

The Pathways Study, a prospective cohort study of women diagnosed with breast cancer, provided the opportunity to examine associations between diet quality and incident CVD-related events, overall, and by different breast cancer treatment types. We hypothesized that higher concordance to healthful eating recommendations would be related to lower risk of CVD and CVD-related death. To our knowledge, this study is the first to evaluate the relationships between diet quality and CVD-specific outcomes in women diagnosed with breast cancer.

## Methods

### Study population

A total of 4504 female breast cancer survivors diagnosed with invasive breast cancer between the years 2005 and 2013 at Kaiser Permanente Northern California (KPNC) were enrolled into the Pathways Study. Details are provided elsewhere (14). Briefly, enrollment occurred an average of 2.3 months (range = 0.7-17.8 months) after diagnosis, and eligibility criteria included female sex; aged 21 years or older; KPNC member; English, Spanish, Cantonese, or Mandarin speaker; living within a 65-mile radius of a field interviewer; diagnosis of incident invasive breast cancer; and no prior history of other invasive cancers. Participants underwent an in-person baseline interview at or around the time of their breast cancer diagnosis and follow-up interviews by mail, phone, internet, or in person.

**Figure 1.**
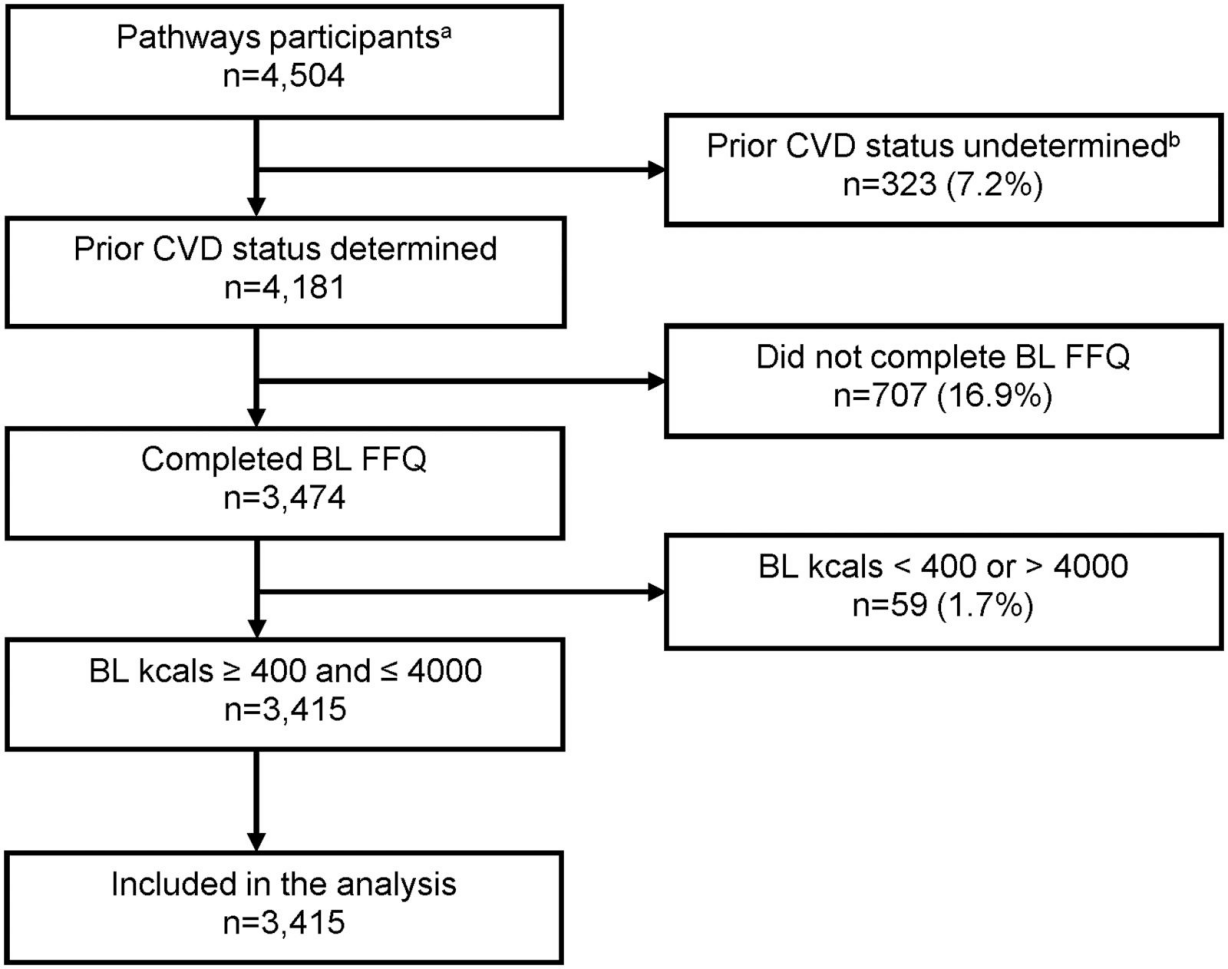
Flowchart of exclusions for participants from the Pathways Study. ^a^Enrolled at or around the time of first invasive breast cancer diagnosis, 2005-2013. ^b^Having a prior CVD event was not determined because of participant having less than 12 months of continuous Kaiser Permanente membership before breast cancer diagnosis. BL = baseline; CVD = cardiovascular disease; FFQ = food frequency questionnaire; kcals = kilocalories.

For this analysis, exclusions included 323 women who had less than 12 months continuous KPNC membership prior to their breast cancer diagnosis, 707 who did not complete the baseline food frequency questionnaire, and 59 with an improbable total energy intake value at baseline (<400 or >4000 kcal/day). The final analytic cohort included 3415 participants (see Figure 1).

This study was approved by the institutional review board of KPNC and participating institutions. Written informed consent was obtained from all participants.

### Dietary assessment

Dietary intake was assessed at baseline using a 139-item modified Block 2005 food frequency questionnaire (15), which ascertained consumption of foods and beverages over the preceding 6 months. From this, 5 previously developed diet quality indices were calculated: Dietary Approaches to Stop Hypertension (DASH) (16), healthy plant-based dietary index (17), 2020 Healthy Eating Index (HEI) (18), American Cancer Society (ACS) nutrition guidelines for cancer prevention (19), and alternate Mediterranean dietary index (20). DASH and alternate Mediterranean dietary index were chosen for their prior associations with decreased hypertension and subsequent decreased CVD (16,21), and the healthy plant-based dietary index, HEI, and ACS nutrition guidelines for cancer prevention were selected because of their associations with improved breast cancer prognosis in prior studies from this cohort (8,9,22). For each index, a higher score indicated greater concordance, suggestive of better diet quality (see Table 1).

**Table 1.** Scoring methods and optimal quantities for each component of Dietary Approaches to Stop Hypertension, healthy plant-based dietary index, 2020 Healthy Eating Index, American Cancer Society nutrition guidelines for cancer prevention, and alternate Mediterranean dietary index

Food group	Dietary Approaches to Stop Hypertension (total = 40 points)	Healthy plant-based dietary index (total = 85 points)	2020 Healthy Eating Index^a^ (total = 100 points)	American Cancer Society nutrition guidelines for cancer prevention (total = 12 points)	Alternate Mediterranean dietary index (total = 8 points)
Dairy					
Low-fat dairy	= Highest quintile				
Total dairy		= Lowest quintile	≥1.3 cups/1000 kcal		
Fats					
Animal fats		= Lowest quintile			
Long-chain fatty acids					
Saturated fats			≤8% of energy		
Trans fats					
Unsaturated fats			Polyunsaturated fats + monounsaturated fats: saturated fats ≥ 2.5		Monounsaturated fats: saturated fats ≥ median
Vegetable oils		= Highest quintile			
Fruits and vegetables					
Fruit					
Fruit juices		= Lowest quintile			
Whole fruits		= Highest quintile	≥0.4 cups/1000 kcal		
Total fruits	= Highest quintile		≥0.8 cups/1000 kcal		≥ Median
Vegetables					
Greens and beans			≥0.2 cups/1000 kcal		
Nonstarchy vegetables^b^	= Highest quintile	= Highest quintile			
Starchy vegetables^c^		= Lowest quintile			
Total vegetables			≥1.1 cups/1000 kcal		≥ Median
Total fruits and vegetables				= Highest quartile^g^	
Grains					
Refined grains		= Lowest quintile	≤1.8 ounces/1000 kcal		
Whole grains	= Highest quintile	= Highest quintile	>1.5 ounces/1000 kcal	= Highest quartile	≥ Median
Proteins					
Eggs		= Lowest quintile			
Legumes^d^		= Highest quintile			≥ Median
Nuts		= Highest quintile			≥ Median
Nuts and legumes^d^	= Highest quintile				
Red and processed meats	= Lowest quintile			= Lowest quartile	< Median
Seafood and fish		= Lowest quintile			≥ Median
Seafood and plant proteins			≥0.8 ounces/1000 kcal		
Total meat^e^		= Lowest quintile			
Total protein			≥2.5 ounces/1000 kcal		
Sodium	= Lowest quintile		≤1.1 grams/1000 kcal		
Sugar					
Added sugar			≤ 6.5% of energy		
Sugar-sweetened beverages^f^	= Lowest quintile	= Lowest quintile			
Sugar-sweetened beverages and highly processed foods and refined grains				= Lowest quartile^h^	
Sweets and desserts		= Lowest quintile			
Teas and coffees		= Highest quintile			

a All amounts per day.

b Does not include potatoes and legumes.

c Does not include sweet potatoes, which are included in nonstarchy vegetables.

d Legumes include beans, peas, soybeans, and soy products such as tofu.

e Includes red and processed meats, poultry, and organ meat.

f Fruit juices not included.

g Half of the score derived from consumption of different types of fruits or vegetables.

h Score split evenly between sugar-sweetened beverages and combined highly processed foods and refined grains.

### DASH score

The DASH score contained 8 dietary components worth 5 points each from cohort-specific quintiles ranging from 1 (lowest) to 5 (highest) (16). Total scores ranged from 0 to 40 by combining points from the favorable components (fruits, vegetables, nuts, grains, and low-fat dairy) and reverse scored adverse components (sodium, red and processed meats, and sugar sweetened beverages) (16).

### Healthy plant-based dietary index score

The healthy plant-based dietary index score ranged from 17 to 85 points and was created from the sum of 17 dietary components, each scored on a 5-point scale from cohort-specific quintiles (17). For 7 of these (vegetable oils, whole fruits, nonstarchy vegetables, whole grains, legumes, nuts, and teas and coffees), greater consumption resulted in a higher healthy plant-based dietary index component score, while for the other 10 (dairy, animal fats, fruit juices, starchy vegetables, refined grains, eggs, seafood and fish, total meat, sweetened beverages, and sweets), greater consumption resulted in a lower component score (17).

### 2020 HEI score

The HEI score, derived from the 2020-2025 Dietary Guidelines for Americans, contained 13 dietary components (18): 6 components (total fruits, whole fruits, total vegetables, greens and beans, total protein foods, and seafood and plant protein foods) were worth up to 5 points each, and 7 others (whole grains, dairy, unsaturated fats, refined grains, sodium, added sugars, and saturated fats) were worth up to 10 points each, for a possible total of 100 points. The HEI components were scored on a density basis (per 1000 kcals or percentage of energy), except for the ratio of unsaturated to saturated fats (18). See Table 1 for further description.

### ACS nutrition guidelines for cancer prevention score

The ACS nutrition guidelines for cancer prevention score reflects the ACS 2020 dietary recommendations for cancer prevention and ranged from 0 to 12 points with 4 food components (19): combined total fruits, vegetables, and legumes (partly rewarding variety of each); whole grains; red and processed meats; and a combined sugar-sweetened beverages, highly processed foods, and refined grains. Each component was given a value on a 4-point scale from 0 (lowest) to 3 (highest) and was based on cohort-specific quartiles for that component. Scores for red and processed meat, sugar-sweetened beverages, highly processed foods, and refined grains were reversed (19).

### Alternate Mediterranean dietary index score

The alternate Mediterranean dietary index score ranged from 0 to 9 and was comprised of 8 dietary components, 7 to be encouraged, and 1 to be moderated. The encouraged components included vegetables, legumes, fruits, nuts, whole grains, seafood, and the ratio of monosaturated to saturated fats, and the moderated component was red and processed meats. Intakes at or above the population median for encouraged components received 1 point, and all other intakes received 0 points. The red and processed meat component was reverse scored (20).

### Other covariables

Self-reported demographic factors including age, race and ethnicity, and education were collected using a baseline questionnaire. Where possible, missing data were supplemented with data obtained from the KPNC electronic health records (EHR). Body mass index and comorbid conditions were obtained from the EHR. An Elixhauser comorbidity index score was calculated from inpatient and ambulatory comorbid conditions dating back 1 year from breast cancer diagnosis (23). Diagnostic and clinical data, including tumor stage; estrogen receptor status; progesterone receptor status; human epidermal growth factor receptor 2 status; type of surgery; and receipt of chemo-, radiation, and endocrine therapies were obtained from the KPNC Cancer Registry and other clinical databases.

### CVD events and mortality

Details are provided elsewhere (24). Briefly, incident CVD outcome events were ascertained from the KPNC EHR using International Classification of Disease diagnostic codes (ninth and tenth revisions) and Current Procedural Terminology codes spanning breast cancer diagnosis through December 31, 2021. Prevalent CVD events used for model-specific exclusions and adjustments were similarly obtained from the earliest available record up to 3 years prior to breast cancer diagnosis. Primary CVD outcomes of interest were ischemic heart disease, heart failure, cardiomyopathy, and stroke. Cardiomyopathy excluded codes for inherited cardiomyopathies (25), and stroke included transient ischemic attack. Secondary CVD outcomes included arrhythmia, cardiac arrest, valvular heart disease, and venous thromboembolic disease.

CVD-related deaths through December 31, 2021, were identified through the KPNC mortality databases, including deaths from the KPNC Cancer Registry; clinical encounters; and linkages with the State of California, Social Security Administration, and National Death Index.

### Statistical analysis

Spearman correlation coefficients were used to compare continuous diet quality index scores, and Pathways Study–specific quartiles for each diet quality index were calculated. Comparisons of baseline characteristics across quartiles of each diet quality index were examined using analysis of variance and Pearson χ^2^ test for categorical variables. Fine and Gray proportional hazards models (26) were used to calculate subdistribution hazard ratios (HRs) and 95% confidence intervals (CIs) to estimate associations between diet quality indices and CVD outcomes. The time scale was defined as the time from breast cancer diagnosis to the first of the following: incident CVD event, death, KPNC disenrollment, or end of study (December 31, 2021).

Participants were excluded from those analyses in which they had the CVD outcome of interest prior to their breast cancer diagnosis and were reincluded for subsequent analyses in which they did not have the CVD outcome of interest prior to their breast cancer diagnosis. Variables included in fully adjusted models were age at breast cancer diagnosis; race and ethnicity; education level; menopausal status; physical activity; smoking; alcohol intake; total energy; cancer stage at diagnosis; estrogen receptor, progesterone receptor, and human epidermal growth factor receptor 2 status; cardiometabolic conditions (including hypertension, dyslipidemia, and diabetes); and any nonoutcome CVD event prior to breast cancer diagnosis. Additional tests for interaction were conducted to assess chemo-, radiation, and endocrine therapies for modification of any observed diet quality associations. Also, mutually adjusted independent associations between individual food components within each diet quality index and any CVD event or CVD-related death were examined. In all models, the quartile representing the lowest diet quality index score was used as the reference group, and trend results corresponding to a 1-unit increase in each index using the noncategorized variable were calculated.

The proportional hazards assumption was assessed using partial likelihood ratio tests comparing the main effects model with models also containing interaction terms for each covariate with time, and evidence for violation was found for menopausal status. We did not, however, observe evidence that diet quality and CVD were modified by menopausal status. Thus, menopausal status was included as a stratification variable for all models, allowing the baseline hazards to vary at each level.

*P* values were 2-sided and considered statistically significant at a value of .05 or less. All statistical analyses were conducted using SAS 9.4 software (SAS Institute, Cary, NC, USA).

## Results

The mean age of participants at diagnosis was 60 years (range = 24-94 years), and women in the highest quartile of DASH compared with the lowest were more likely to be older, White, more educated, physically active, postmenopausal, and nonsmokers and have lower body mass index and fewer comorbidities, as well as have higher reported energy intake at baseline. They were also more likely to have a lower cancer stage at diagnosis, to have received radiation therapy, and to have had a secondary CVD event prior to their breast cancer diagnosis and were less likely to have received chemotherapy (Table 2). Differences in participant characteristics across categories of other diet quality indices were qualitatively similar to DASH. Although Spearman correlation coefficients comparing the diet quality indices suggested some similarities among them, each appears to assess unique components of diet (Supplementary Table 1, available online).

**Table 2.** Baseline characteristics of the Pathways Heart Study participants across quartiles of Dietary Approaches to Stop Hypertension (n = 3415)

	Total (n = 3415)	Quartiles of Dietary Approaches to Stop Hypertension				*P*
		Q1 (n = 768)	Q2 (n = 1069)	Q3 (n = 749)	Q4 (n = 829)	
Range	10-37	10-20	21-24	25-27	28-37	
Characteristics, mean (SD)						
Age at diagnosis, y	60.1 (11.9)	57.7 (12.1)	59.5 (11.4)	61.2 (12.1)	62.2 (11.6)	<.001^b^
Physical activity, metabolic equivalent of task h/wk^a^	53.8 (36.0)	42.7 (30.2)	51.4 (34.3)	55.5 (34.3)	65.7 (40.5)	<.001^b^
Body mass index, kg/m^2^	28.5 (6.7)	29.7 (7.4)	29.2 (6.9)	28.1 (6.2)	26.8 (5.8)	<.001^b^
Alcohol intake, g/d	7.2 (13.0)	7.5 (14.5)	7.4 (13.6)	7.4 (12.6)	6.5 (10.9)	.40^b^
Energy intake, kcal/d	1463.4 (566.2)	1397.7 (570.8)	1420.6 (586.9)	1480.8 (575.9)	1563.6 (509.5)	<.001^b^
Health plan utilization, visits	3.1 (3.5)	3.1 (3.0)	3.2 (3.7)	3.1 (4.0)	3.0 (3.3)	.71^b^
Elixhauser comorbidity score	1.3 (1.6)	1.5 (1.8)	1.4 (1.6)	1.3 (1.5)	1.2 (1.4)	<.001^b^
Characteristics, No. (%)						
Race and ethnicity						<.001^c^
American Indian or Alaska Native	70 (2.0)	17 (2.2)	24 (2.2)	17 (2.3)	12 (1.4)	
Asian or Pacific Islander	439 (12.9)	142 (18.5)	135 (12.6)	85 (11.3)	77 (9.3)	
Black	214 (6.3)	91 (11.8)	69 (6.5)	28 (3.7)	26 (3.1)	
Hispanic	346 (10.1)	81 (10.5)	134 (12.5)	69 (9.2)	62 (7.5)	
White	2346 (68.7)	437 (56.9)	707 (66.1)	550 (73.4)	652 (78.6)	
Education						<.001^c^
High school or less	499 (14.6)	168 (21.9)	181 (16.9)	83 (11.1)	67 (8.1)	
Some college	1162 (34.0)	288 (37.5)	388 (36.3)	261 (34.8)	225 (27.1)	
College graduate	964 (28.2)	216 (28.1)	282 (26.4)	206 (27.5)	260 (31.4)	
Postgraduate	788 (23.1)	96 (12.5)	218 (20.4)	198 (26.4)	276 (33.3)	
Unknown	2 (0.1)	0 (0.0)	0 (0.0)	1 (0.1)	1 (0.1)	
Menopausal status						<.001^c^
Premenopausal	943 (27.6)	265 (34.5)	310 (29.0)	187 (25.0)	181 (21.8)	
Postmenopausal	2472 (72.4)	503 (65.5)	759 (71.0)	562 (75.0)	648 (78.2)	
Smoking status						<.001^c^
Never	1952 (57.2)	404 (52.6)	610 (57.1)	444 (59.3)	494 (59.6)	
Former	1319 (38.6)	303 (39.5)	414 (38.7)	282 (37.7)	320 (38.6)	
Current	138 (4.0)	61 (7.9)	45 (4.2)	21 (2.8)	11 (1.3)	
Unknown	6 (0.2)	0 (0.0)	0 (0.0)	2 (0.3)	4 (0.5)	
Cancer stage						.01^c^
I	1883 (55.1)	415 (54.0)	548 (51.3)	419 (55.9)	501 (60.4)	
II	1163 (34.1)	264 (34.4)	401 (37.5)	252 (33.6)	246 (29.7)	
III	319 (9.3)	74 (9.6)	102 (9.5)	67 (8.9)	76 (9.2)	
IV	50 (1.5)	15 (2.0)	18 (1.7)	11 (1.5)	6 (0.7)	
Estrogen receptor status						.56^c^
Positive	2870 (84.0)	635 (82.7)	898 (84.0)	629 (84.0)	708 (85.4)	
Negative	543 (15.9)	132 (17.2)	170 (15.9)	120 (16.0)	121 (14.6)	
Unknown	2 (0.1)	1 (0.1)	1 (0.1)	0 (0.0)	0 (0.0)	
Progesterone receptor status						.59^c^
Positive	2199 (64.4)	503 (65.5)	694 (64.9)	483 (64.5)	519 (62.6)	
Negative	1212 (35.5)	262 (34.1)	374 (35.0)	266 (35.5)	310 (37.4)	
Unknown	4 (0.1)	3 (0.4)	1 (0.1)	0 (0.0)	0 (0.0)	
Human epidermal growth factor receptor 2 status						.76^c^
Positive	439 (12.9)	109 (14.2)	139 (13.0)	93 (12.4)	98 (11.8)	
Negative	2842 (83.2)	634 (82.6)	885 (82.8)	624 (83.3)	699 (84.3)	
Unknown, not done	134 (3.9)	25 (3.3)	45 (4.2)	32 (4.3)	32 (3.9)	
Surgery type						.13^c^
Lumpectomy	2042 (59.8)	445 (57.9)	631 (59.0)	459 (61.3)	507 (61.2)	
Mastectomy	1270 (37.2)	303 (39.5)	399 (37.3)	262 (35.0)	306 (36.9)	
None	101 (3.0)	20 (2.6)	39 (3.6)	27 (3.6)	15 (1.8)	
Unknown	2 (0.1)	0 (0.0)	0 (0.0)	1 (0.1)	1 (0.1)	
Chemotherapy						<.001^c^
No	1834 (53.7)	377 (49.1)	534 (50.0)	431 (57.5)	492 (59.3)	
Yes	1570 (46.0)	390 (50.8)	528 (49.4)	317 (42.3)	335 (40.4)	
Unknown	11 (0.3)	1 (0.1)	7 (0.7)	1 (0.1)	2 (0.2)	
Radiation therapy						.01^c^
No	1890 (55.3)	460 (59.9)	595 (55.7)	404 (53.9)	431 (52.0)	
Yes	1525 (44.7)	308 (40.1)	474 (44.3)	345 (46.1)	398 (48.0)	
Hormonal therapy						.82^c^
No	836 (24.5)	188 (24.5)	252 (23.6)	191 (25.5)	205 (24.7)	
Yes	2555 (74.8)	571 (74.3)	810 (75.8)	553 (73.8)	621 (74.9)	
Unknown	24 (0.7)	9 (1.2)	7 (0.7)	5 (0.7)	3 (0.4)	
Prior primary CVD event^d^						.33^c^
No	3184 (93.2)	705 (91.8)	1003 (93.8)	699 (93.3)	777 (93.7)	
Yes	231 (6.8)	63 (8.2)	66 (6.2)	50 (6.7)	52 (6.3)	
Prior secondary CVD event^e^						.048^c^
No	3069 (89.9)	707 (92.1)	966 (90.4)	665 (88.8)	731 (88.2)	
Yes	346 (10.1)	61 (7.9)	103 (9.6)	84 (11.2)	98 (11.8)	
Prior cardiometabolic condition^f^						.27^c^
No	1529 (44.8)	329 (42.8)	471 (44.1)	335 (44.7)	394 (47.5)	
Yes	1886 (55.2)	439 (57.2)	598 (55.9)	414 (55.3)	435 (52.5)	

a Moderate to vigorous physical activity. Four participants with missing values. CVD = cardiovascular disease; Q = quartile.

b The *P* value is from analysis of variance.

c The *P* value is from the Pearson χ^2^ test.

d Primary CVD events prior to breast cancer diagnosis include ischemic heart disease, heart failure, cardiomyopathy, and stroke and/or transient ischemic attack.

e Secondary CVD events prior to breast cancer diagnosis include arrhythmia, cardiac arrest, valvular heart disease, and venous thromboembolic disease.

f Cardiometabolic conditions prior to breast cancer diagnosis include diabetes, dyslipidemia, and hypertension.

### Diet quality indices and study outcomes

Food frequency questionnaires were completed an average of 2.2 months (range = 0.3-7.6 months) after a breast cancer diagnosis, and a total 650 (19.0%) incident CVD events and 341 (10.0%) CVD-related deaths were ascertained over 39 292 person-years of follow-up (mean = 11.5, standard deviation = 3.6 years). For primary CVD events, participants in the highest quartiles of the DASH and healthy plant-based dietary indices had lower risks of heart failure (DASH: HR = 0.53, 95% CI = 0.33 to 0.87; *P*_trend_ = .03; healthy plant-based dietary index: HR = 0.60, 95% CI = 0.39 to 0.94; *P*_trend_ = .02) compared with participants in the lowest quartile (Table 3).

**Table 3.** Subdistribution hazard ratios and 95% confidence intervals for quartiles of baseline diet quality on primary CVD events after breast cancer diagnosis^a^^,^^b^

Diet quality indices	Ischemic heart disease (n = 3240)				Heart failure (n = 3327)				Cardiomyopathy (n = 3377)				Stroke and/or transient ischemic attack (n = 3370)			
	Events	Person-time, y	HR (95% CI)	*P*_trend_	Events	Person-time, y	HR (95% CI)	*P*_trend_	Events	Person-time, y	HR (95% CI)	*P*_trend_	Events	Person-time, y	HR (95% CI)	*P*_trend_
Dietary Approaches to Stop Hypertension				.96				.03				.27				.27
Q1	36	7931	Referent		52	8189	Referent		19	8396	Referent		29	8396	Referent	
Q2	51	11 381	1.06 (0.68 to 1.64)		68	11 806	0.88 (0.61 to 1.29)		18	12 020	0.81 (0.41 to 1.58)		35	11 991	0.82 (0.49 to 1.37)	
Q3	32	7985	1.02 (0.61 to 1.72)		46	8191	0.81 (0.53 to 1.24)		10	8341	0.68 (0.30 to 1.56)		22	8363	0.61 (0.34 to 1.10)	
Q4	31	9033	0.95 (0.56 to 1.60)		33	9308	0.53 (0.33 to 0.87)		6	9574	0.41 (0.15 to 1.10)		27	9457	0.74 (0.43 to 1.27)	
Healthy plant-based dietary index				.44				.02				.61				.55
Q1	36	8666	Referent		59	8910	Referent		18	9090	Referent		27	9118	Referent	
Q2	44	9999	1.36 (0.85 to 2.17)		51	10 307	0.80 (0.53 to 1.20)		15	10 498	0.90 (0.45 to 1.81)		38	10 475	1.18 (0.69 to 2.02)	
Q3	29	8492	0.97 (0.59 to 1.61)		49	8730	0.85 (0.57 to 1.28)		8	8974	0.51 (0.21 to 1.23)		25	8924	1.04 (0.56 to 1.92)	
Q4	41	9172	1.44 (0.89 to 2.35)		40	9546	0.60 (0.39 to 0.94)		12	9769	0.89 (0.38 to 2.09)		23	9689	0.84 (0.46 to 1.53)	
2020 Healthy Eating Index				.27				.29				.67				.98
Q1	34	8644	Referent		47	8939	Referent		16	9178	Referent		31	9094	Referent	
Q2	36	9265	1.09 (0.67 to 1.75)		64	9435	1.32 (0.89 to 1.97)		15	9636	0.89 (0.43 to 1.86)		25	9685	0.80 (0.46 to 1.37)	
Q3	41	9769	1.30 (0.80 to 2.09)		46	10 169	0.90 (0.58 to 1.37)		12	10 321	0.83 (0.36 to 1.92)		22	10 348	0.64 (0.36 to 1.12)	
Q4	39	8651	1.42 (0.87 to 2.33)		42	8950	0.84 (0.53 to 1.33)		10	9196	0.87 (0.37 to 2.05)		35	9080	1.07 (0.65 to 1.77)	
American Cancer Society nutrition guidelines for cancer prevention				.29				.74				.29				.29
Q1	35	9194	Referent		49	9471	Referent		20	9637	Referent		34	9570	Referent	
Q2	43	9436	1.25 (0.79 to 1.99)		48	9771	1.00 (0.66 to 1.52)		14	9979	0.77 (0.37 to 1.59)		27	10 032	0.66 (0.39 to 1.14)	
Q3	34	8805	1.08 (0.66 to 1.77)		59	9053	1.24 (0.84 to 1.84)		13	9270	0.80 (0.39 to 1.66)		27	9275	0.74 (0.43 to 1.25)	
Q4	38	8894	1.35 (0.83 to 2.20)		43	9198	1.02 (0.66 to 1.58)		6	9446	0.41 (0.15 to 1.09)		25	9329	0.70 (0.41 to 1.21)	
Alternate Mediterranean dietary index				.53				.13				.12				.77
Q1	32	8174	Referent		51	8524	Referent		17	8742	Referent		33	8612	Referent	
Q2	26	6405	1.21 (0.70 to 2.09)		44	6607	1.12 (0.73 to 1.73)		12	6720	1.06 (0.48 to 2.32)		21	6744	0.78 (0.43 to 1.40)	
Q3	59	12 877	1.41 (0.87 to 2.29)		76	13 230	1.21 (0.82 to 1.80)		18	13 602	0.85 (0.41 to 1.75)		33	13 634	0.68 (0.40 to 1.16)	
Q4	33	8873	1.27 (0.71 to 2.26)		28	9133	0.68 (0.39 to 1.19)		6	9268	0.55 (0.18 to 1.66)		26	9217	0.89 (0.51 to 1.56)	

a Adjusted for age at diagnosis, race and ethnicity, education level, menopausal status, physical activity, smoking status, energy intake, alcohol intake, health plan utilization, comorbidities, cancer stage, estrogen receptor status, progesterone receptor status, human epidermal growth factor receptor 2 status, cardiometabolic conditions, and any nonoutcome CVD event prior to breast cancer diagnosis. CI = confidence interval; CVD = cardiovascular disease; HR = hazard ratio; Q = quartile.

b Participants with CVD outcome of interest prior to breast cancer diagnosis were excluded.

For secondary CVD events, participants in the highest quartile of the DASH diet quality index had lower risks of arrhythmia (HR = 0.77, 95% CI = 0.62 to 0.94: *P*_trend_ = .008), cardiac arrest (HR = 0.77, 95% CI = 0.61 to 0.96; *P*_trend_ = .02), valvular heart disease (HR = 0.79, 95% CI = 0.64 to 0.98; *P*_trend_ = .046), and venous thromboembolic disease (HR = 0.75, 95% CI = 0.60 to 0.93; *P*_trend_ = .01), when comparing participants in the highest quartile with those in the lowest. Additionally, participants in the highest quartile of the alternate Mediterranean dietary quality index had a lower risk of arrhythmia (HR = 0.74, 95% CI = 0.60 to 0.93; *P*_trend_ = .02) when compared with participants in the lowest quartile (Table 4).

**Table 4.** Subdistribution hazard ratios and 95% confidence intervals for quartiles of baseline diet quality on secondary CVD events after breast cancer diagnosis^a^^,^^b^

Diet quality indices	Arrhythmia (n = 3149)				Cardiac arrest (n = 3401)				Valvular heart disease (n = 3344)				Venous thromboembolic disease (n = 3318)			
	Events	Person-time, y	HR (95% CI)	*P*_trend_	Events	Person-time, y	HR (95% CI)	*P*_trend_	Events	Person-time, y	HR (95% CI)	*P*_trend_	Events	Person-time, y	HR (95% CI)	*P*_trend_
Dietary Approaches to Stop Hypertension				.008				.02				.046				.01
Q1	81	7748	Referent		34	8546	Referent		20	8387	Referent		44	8162	Referent	
Q2	111	10 866	0.90 (0.76 to 1.08)		36	12 167	0.92 (0.76 to 1.10)		26	11 919	0.91 (0.76 to 1.09)		57	11 584	0.89 (0.75 to 1.07)	
Q3	79	7422	0.85 (0.70 to 1.03)		14	8485	0.90 (0.73 to 1.10)		21	8248	0.89 (0.73 to 1.10)		36	8,079	0.88 (0.72 to 1.08)	
Q4	86	8485	0.77 (0.62 to 0.94)		13	9604	0.77 (0.61 to 0.96)		25	9325	0.79 (0.64 to 0.98)		39	9078	0.75 (0.60 to 0.93)	
Healthy plant-based dietary index				.05				.24				.24				.24
Q1	91	8293	Referent		34	9269	Referent		21	9079	Referent		44	8775	Referent	
Q2	92	9559	1.02 (0.85 to 1.22)		24	10 646	1.08 (0.89 to 1.31)		17	10 502	1.06 (0.87 to 1.28)		48	10 235	1.01 (0.84 to 1.22)	
Q3	86	7885	1.12 (0.92 to 1.35)		19	9027	1.03 (0.84 to 1.27)		30	8731	1.10 (0.90 to 1.34)		42	8583	0.98 (0.80 to 1.20)	
Q4	88	8784	0.90 (0.73 to 1.10)		20	9859	0.93 (0.75 to 1.15)		24	9568	0.91 (0.73 to 1.12)		42	9310	0.92 (0.75 to 1.13)	
2020 Healthy Eating Index				.10				.29				.64				.42
Q1	91	8301	Referent		34	9282	Referent		20	9097	Referent		35	8897	Referent	
Q2	83	8812	0.85 (0.71 to 1.02)		25	9818	0.91 (0.75 to 1.10)		23	9579	0.95 (0.79 to 1.15)		52	9398	0.91 (0.75 to 1.10)	
Q3	94	9275	0.91 (0.76 to 1.09)		23	10 443	0.97 (0.79 to 1.17)		19	10 273	0.95 (0.79 to 1.16)		41	9968	0.97 (0.80 to 1.17)	
Q4	89	8132	0.86 (0.70 to 1.04)		15	9259	0.90 (0.73 to 1.10)		30	8931	0.95 (0.78 to 1.16)		48	8640	0.93 (0.76 to 1.13)	
American Cancer Society nutrition guidelines for cancer prevention				.02				.11				.17				.03
Q1	101	8794	Referent		40	9778	Referent		17	9607	Referent		55	9329	Referent	
Q2	86	9027	0.83 (0.69 to 0.99)		19	10 118	0.87 (0.72 to 1.05)		21	9910	0.91 (0.76 to 1.10)		41	9594	0.90 (0.75 to 1.07)	
Q3	84	8254	0.93 (0.78 to 1.12)		22	9423	1.00 (0.83 to 1.21)		33	9138	1.04 (0.86 to 1.25)		35	9066	0.94 (0.78 to 1.13)	
Q4	86	8447	0.82 (0.68 to 1.00)		16	9483	0.83 (0.67 to 1.02)		21	9224	0.85 (0.69 to 1.05)		45	8913	0.82 (0.67 to 1.00)	
Alternate Mediterranean dietary index				.02				.12				.24				.13
Q1	88	7948	Referent		31	8840	Referent		18	8684	Referent		43	8434	Referent	
Q2	66	6130	1.01 (0.83 to 1.23)		19	6861	0.99 (0.80 to 1.22)		19	6699	1.04 (0.85 to 1.29)		34	6522	1.01 (0.82 to 1.24)	
Q3	134	12 183	1.01 (0.85 to 1.21)		36	13 768	1.04 (0.86 to 1.25)		35	13 383	1.09 (0.91 to 1.31)		69	13 118	1.04 (0.87 to 1.25)	
Q4	69	8261	0.74 (0.60 to 0.93)		11	9332	0.78 (0.61 to 0.99)		20	9113	0.81 (0.64 to 1.03)		30	8828	0.78 (0.62 to 0.99)	

a Adjusted for age at diagnosis, race and ethnicity, education level, menopausal status, physical activity, smoking status, energy intake, alcohol intake, health plan utilization, comorbidities, cancer stage, estrogen receptor status, progesterone receptor status, human epidermal growth factor receptor 2 status, cardiometabolic conditions, and any nonoutcome CVD event prior to breast cancer diagnosis. CI = confidence interval; CVD = cardiovascular disease; HR = hazard ratio; Q = quartile.

b Participants with CVD outcome of interest prior to breast cancer diagnosis were excluded.

Participants in the highest quartile of the DASH diet quality index had a lower risk of CVD-related death relative to those in the lowest (HR = 0.70, 95% CI = 0.50 to 0.99; *P*_trend_ = .04). Also, the strongest, most consistent linear dose-response association was observed across quartiles of the DASH diet quality index and any CVD event (Q4 vs Q1: HR = 0.68, 95% CI = 0.53 to 0.87; Q3 vs Q1: HR = 0.78, 95% CI = 0.61 to 0.99; Q2 vs Q1: HR = 0.79, 95% CI = 0.64 to 0.98; *P*_trend_ = .03) (Table 5).

**Table 5.** Subdistribution hazard ratios and 95% confidence intervals for quartiles of baseline diet quality on CVD-related death and composite CVD outcomes after breast cancer diagnosis^a^^,^^b^

Diet quality indices	Any CVD event (n = 2906)				CVD-related death (n = 3402)				Any CVD event or CVD-related death (n = 2906)			
	Events	Person-time, y	HR (95% CI)	*P*_trend_	Events	Person-time, y	HR (95% CI)	*P*_trend_	Events	Person-time, y	HR (95% CI)	*P*_trend_
Dietary Approaches to Stop Hypertension				.03				.04				.05
Q1	162	6677.1	Referent		91	8685.6	Referent		173	6677.1	Referent	
Q2	197	9631.3	0.79 (0.64 to 0.98)		106	12 275.1	0.86 (0.64 to 1.17)		211	9631.3	0.79 (0.64 to 0.97)	
Q3	137	6590.8	0.78 (0.61 to 0.99)		69	8517.8	0.68 (0.49 to 0.96)		147	6590.8	0.78 (0.62 to 0.99)	
Q4	131	7638.4	0.68 (0.53 to 0.87)		74	9665.6	0.70 (0.50 to 0.99)		146	7638.4	0.72 (0.56 to 0.91)	
Healthy plant-based dietary index				.12				.26				.27
Q1	164	7244.2	Referent		84	9385.8	Referent		173	7244.2	Referent	
Q2	180	8534.1	0.98 (0.78 to 1.22)		101	10 755.4	1.06 (0.77 to 1.45)		195	8534.1	1.03 (0.83 to 1.28)	
Q3	151	7010.5	0.99 (0.78 to 1.25)		76	9073.4	0.97 (0.69 to 1.36)		163	7010.5	1.05 (0.83 to 1.31)	
Q4	132	7748.8	0.82 (0.64 to 1.05)		79	9929.6	0.90 (0.64 to 1.27)		146	7748.8	0.90 (0.71 to 1.13)	
2020 Healthy Eating Index				.55				.09				.55
Q1	158	7261.4	Referent		97	9413.5	Referent		173	7261.4	Referent	
Q2	164	7926.9	0.94 (0.75 to 1.17)		85	9913.5	0.84 (0.62 to 1.13)		174	7926.9	0.90 (0.73 to 1.11)	
Q3	152	8212.1	0.84 (0.67 to 1.06)		70	10 508.3	0.68 (0.49 to 0.93)		165	8212.1	0.83 (0.66 to 1.03)	
Q4	153	7137.1	0.98 (0.78 to 1.24)		88	9308.9	0.83 (0.61 to 1.13)		165	7137.1	0.97 (0.77 to 1.21)	
American Cancer Society nutrition guidelines for cancer prevention				.01				.10				.03
Q1	190	7690.3	Referent		108	9899.8	Referent		200	7690.3	Referent	
Q2	152	8091.2	0.73 (0.59 to 0.91)		80	10 251.2	0.67 (0.49 to 0.91)		167	8091.2	0.77 (0.62 to 0.96)	
Q3	149	7256	0.78 (0.62 to 0.98)		82	9460.6	0.79 (0.59 to 1.07)		163	7256	0.81 (0.65 to 1.01)	
Q4	136	7500.1	0.76 (0.61 to 0.96)		70	9532.6	0.70 (0.51 to 0.97)		147	7500.1	0.80 (0.64 to 0.99)	
Alternate Mediterranean dietary index				.07				.23				.09
Q1	170	6814.8	Referent		102	8963.3	Referent		182	6814.8	Referent	
Q2	118	5421.1	0.93 (0.73 to 1.18)		58	6953.1	0.83 (0.59 to 1.17)		124	5421.1	0.91 (0.72 to 1.15)	
Q3	218	10 856.9	0.88 (0.71 to 1.10)		126	13 853.1	1.02 (0.77 to 1.36)		240	10 856.9	0.92 (0.74 to 1.13)	
Q4	121	7444.7	0.73 (0.56 to 0.96)		54	9374.7	0.76 (0.52 to 1.10)		131	7444.7	0.73 (0.56 to 0.95)	

a Adjusted for age at diagnosis, race and ethnicity, education level, menopausal status, physical activity, smoking status, energy intake, alcohol intake, health plan utilization, comorbidities, cancer stage, estrogen receptor status, progesterone receptor status, human epidermal growth factor receptor 2 status, cardiometabolic conditions, and any nonoutcome CVD event prior to breast diagnosis. CI = confidence interval; CVD = cardiovascular disease; HR = hazard ratio; Q = quartile.

b Participants with CVD outcome of interest prior to breast diagnosis were excluded.

### Breast cancer treatment status

The relationship between the DASH diet quality index and any CVD event or CVD-related death appeared to be modified by chemotherapy treatment type (*P*_interaction_* *=* *.03). Among women using anthracycline with or without trastuzumab, those with diets more concordant with the DASH diet quality index had a lower risk of any CVD event or CVD-related death as compared with those least concordant, which was not observed among women using other types of chemotherapies. For women using anthracycline without trastuzumab, those in the highest quartile of the DASH diet quality index had a lower risk of any CVD event or CVD-related death as compared with those in the lowest quartile, though the test for trend was not statistically significant (HR = 0.57, 95% CI = 0.34 to 0.94; *P*_trend_* *=* *.16). For women using anthracycline with trastuzumab, those with diets more concordant to the DASH diet quality index had a lower risk of any CVD event or CVD-related death as compared with those who were less concordant (HR = 0.20, 95% CI = 0.04 to 1.00; *P*_trend_* *=* *.047) (Supplementary Table 2, available online).

There was no evidence of modification by radiation status, however, we found statistically significant tests for trend between DASH (*P*_trend_* *=* *.02) and ACS nutrition guidelines for cancer prevention (*P*_trend_* *=* *.03) and any CVD event or CVD-related death among participants who did not receive radiation therapy, and no statistically significant associations among women who did (Supplementary Table 3, available online). Also, tamoxifen use appeared to modify the relationship between HEI and any CVD event or CVD-related death (*P*_interaction_ = .01) (Supplementary Table 4, available online).

### Individual food components

Mutually adjusted associations between individual food components and CVD outcomes can be found in Supplementary Table 5 (available online). Most notable was higher consumption of low-fat dairy in the case of DASH (HR = 0.85, 95% CI = 0.79 to 0.93) and total dairy in the case of HEI (HR = 0.93, 95% CI = 0.88 to 0.98) being associated with lower risk of CVD-related death. Likewise, lower consumption of total dairy in the case of the healthy plant-based dietary index was associated with higher risk of CVD-related death (HR = 1.13, 95% CI = 1.03 to 1.23). Other notable associations were lower consumption of eggs (HR = 1.09, 95% CI = 1.02 to 1.16) and seafood (HR = 1.08, 95% CI = 1.01 to 1.15) being associated with higher risk of having any CVD event and higher consumption of legumes (HR = 0.93, 95% CI = 0.87 to 0.99) and lower consumption of total meat (HR = 0.91, 95% CI = 0.85 to 0.98) being associated with lower risk of having any CVD event, in the case of the healthy plant-based dietary index. Also, in the case of the alternate Mediterranean dietary index, lower consumption of red and processed meats was associated with lower risk of having any CVD event (HR = 0.82, 95% CI = 0.68 to 0.98), and in the case of the ACS nutrition guidelines for cancer prevention, higher consumption of fruits and vegetables was associated with lower risk of CVD-related death (HR = 0.83, 95% CI = 0.71 to 0.98).

## Discussion

In this prospective cohort study of 3415 breast cancer survivors, participants who reported dietary patterns that were highly concordant with the DASH diet quality index were at lower risk of heart failure, arrhythmia, cardiac arrest, valvular heart disease, and venous thromboembolic disease, as well as CVD-related death. Other notable associations observed were between the healthy plant-based dietary index and heart failure, as well as the alternate Mediterranean dietary index and arrhythmia. No clear patterns emerged when examining the associations between HEI or ACS nutrition guidelines for cancer prevention and any specific CVD-related outcome, though higher concordance with ACS nutrition guidelines for cancer prevention was associated with lower risk of having any CVD event.

We also examined the mutually adjusted food components within each diet quality index on CVD. The most consistently associated food component was that of higher dairy consumption (low-fat dairy in the case of DASH and total dairy in the cases of the healthy plant-based dietary index and HEI) being associated with lower risk of CVD-related death. These findings are consistent with a recent meta-analysis suggesting that consumption of total dairy (low and high fat) reduces the risk of hypertension, coronary heart disease, and stroke within the general population and may be an important consideration for both clinicians and breast cancer patients (27).

Women in our study whose diets were more concordant with the DASH diet quality index and who received anthracycline with or without trastuzumab, had a lower risk of any CVD event or CVD-related death as compared with women whose diets were less concordant. We previously reported in the Pathways Heart Study that women who received anthracycline without trastuzumab had nearly 2 times higher risk, and women who received anthracycline with trastuzumab had more than 3 times higher risk of heart failure and/or cardiomyopathy as compared with matched participants without a history of breast cancer (24). These new results presented herein may provide clinicians with important dietary guidance for women undergoing these specific chemotherapy regimens. As our findings from diet quality on CVD by radiation and endocrine therapies were inconsistent, further investigation is recommended.

Although the DASH, healthy plant-based, and alternate Mediterranean diet quality indices differ in some of its components, all are characterized by high consumption of fruits and vegetables, whole grains, nuts, and legumes and low consumption of red and processed meats, and each have been shown to lower the risk of heart disease in the general population (16,17,21). The DASH and Mediterranean diets are vetted eating patterns designed to prevent and treat hypertension as well as CVD (21,28), and each have numerous resources already in place for patients (29). To our knowledge, this is the first study to examine the associations between these dietary patterns and CVD outcomes in breast cancer survivors.

This study has several strengths, including a large sample drawn from a population of women newly diagnosed with invasive breast cancer and prospective longitudinal data collection with a long follow-up period and comprehensive measures of dietary exposures, outcomes, and covariates. Despite these strengths, this approach does not directly address limitations arising from measurement error of dietary intakes, which can result in biased estimates and exposure misclassification (30). Furthermore, we recognize this study uses 1 dietary measurement at a single time point, which approximately reflects the 6-month period prior to baseline and does not take in account any dietary changes that may have occurred after that time. Also, diet quality index scores are generally limited by the subjectivity of their specified groupings (foods may be grouped or weighted differently across indices), by the dependence on their underlying guidelines, and the potential for classifying individuals into broad categories that may not reveal the nuances of a healthy diet (31). Women with breast cancer who enrolled in this study might also be systematically different from those who did not. However, other data showed that the Pathways Study cohort reflects the eligible underlying population, with minor shifts toward slightly younger ages, higher proportion of White women, and earlier stages at diagnosis.

In summary, implementing diets concordant with healthy dietary patterns may be beneficial for preventing CVD and CVD-related deaths for breast cancer patients, who have been shown to be at higher risk of CVD as compared with the general population. Additionally, the DASH diet may provide the most benefit, particularly for those breast cancer patients receiving cardiotoxic chemotherapies.

## Supplementary Material

pkae013_Supplementary_Data

## Data Availability

The data underlying this article are available and accessible through the procedures outlined on the study website at https://divisionofresearch.kaiserpermanente.org/research/pathways/for-researchers/.
