# Supplementary material for: Diet quality and cardiovascular disease risk among breast cancer survivors in the Pathways Study
Source: JNCI Cancer Spectr. 2024 Apr 17;8(2):pkae013. doi: 10.1093/jncics/pkae013 (PMC11021810; doi:10.1093/jncics/pkae013)
Supplement: pkae013_Supplementary_Data [file pkae013_supplementary_data.pdf]

Supplementary Table 1. Spearman correlation coefficients among baseline total summary scores for DASH, hPDI, HEI, ACS and aMED (n=3,415).[a]

	DASH	hPDI	HEI	ACS	aMED
DASH	1.00				
hPDI	0.64	1.00			
HEI	0.74	0.54	1.00		
ACS	0.60	0.70	0.57	1.00	
aMED	0.66	0.38	0.58	0.27	1.00

Abbreviations: DASH (Dietary Approaches to Stop Hypertension score), hPDI (healthy plant-based index score), HEI (Healthy Eating Index score), ACS (American Cancer Society nutrition guidelines score), aMED (alternate Mediterranean Diet Index score)

[a]All $p < .001$.

Supplementary Table 2. Subdistribution hazard ratios and 95% confidence intervals for quartiles of baseline diet quality on any CVD event or CVD-related death after breast cancer diagnosis, by chemotherapy type (n=2,906)[a,b,c]

	Anthracycline without trastuzumab (n=814)				Anthracycline with trastuzumab (n=156)				Trastuzumab without anthracycline (n=159)				Cyclophosphamide, fluoropyrimidine & taxanes (n=350)				
DQI	Ev	Pt (yrs)	HR (95% CI)	P_{tr}	Ev	Pt (yrs)	HR (95% CI)	P_{tr}	Ev	Pt (yrs)	HR (95% CI)	P_{tr}	Ev	Pt (yrs)	HR (95% CI)	P_{tr}	P_{int}[d]
DASH				.16				.047				.80				.43	.03
Q1	50	2,027	ref		12	428	ref		6	349	ref		17	812	ref		
Q2	68	3,035	0.83 (0.56, 1.23)		16	556	1.49 (0.44, 4.96)		9	468	0.94 (0.33, 2.71)		25	1,092	0.83 (0.37, 1.86)		
Q3	42	1,623	1.04 (0.66, 1.63)		8	390	0.42 (0.10, 1.82)		6	366	1.18 (0.31, 4.45)		19	675	1.16 (0.49, 2.76)		
Q4	28	1,829	0.57 (0.34, 0.94)		3	358	0.20 (0.04, 1.00)		8	408	0.65 (0.20, 2.13)		11	884	0.45 (0.16, 1.30)		
hPDI				.77				.21				.35				.32	.77
Q1	49	2,381	ref		12	362	ref		9	501	ref		17	876	ref		
Q2	59	2,638	1.22 (0.81, 1.85)		12	521	0.63 (0.22, 1.78)		6	294	1.17 (0.36, 3.86)		24	949	1.45 (0.65, 3.21)		
Q3	43	1,771	1.33 (0.85, 2.09)		11	432	1.03 (0.35, 3.02)		6	402	0.30 (0.08, 1.13)		18	841	0.90 (0.38, 2.13)		
Q4	37	1,725	1.10 (0.67, 1.79)		4	416	0.34 (0.08, 1.41)		8	395	0.61 (0.17, 2.19)		13	796	0.66 (0.26, 1.71)		
HEI				.45				.26				.45				.34	.51
Q1	46	2,229	ref		14	510	ref		8	378	ref		22	917	ref		
Q2	58	2,338	1.03 (0.68, 1.55)		13	456	1.32 (0.43, 4.06)		6	397	0.77 (0.22, 2.71)		20	946	0.74 (0.37, 1.47)		
Q3	52	2,176	1.02 (0.66, 1.57)		8	441	0.94 (0.23, 3.95)		7	406	0.92 (0.28, 2.99)		17	904	0.63 (0.30, 1.29)		
Q4	32	1,771	0.76 (0.46, 1.25)		4	325	0.46 (0.08, 2.54)		8	411	0.64 (0.21, 1.95)		13	697	0.58 (0.23, 1.51)		
ACS				.04				.80				.77				.11	.93
Q1	62	2,591	ref		13	465	ref		9	415	ref		24	903	ref		
Q2	48	2,304	0.73 (0.50, 1.08)		12	479	1.21 (0.36, 4.10)		5	375	0.47 (0.11, 1.95)		13	860	0.57 (0.27, 1.20)		
Q3	42	1,826	0.74 (0.47, 1.15)		9	487	0.97 (0.28, 3.37)		5	357	0.36 (0.08, 1.58)		22	797	0.68 (0.31, 1.46)		
Q4	36	1,793	0.70 (0.45, 1.07)		5	300	1.25 (0.34, 4.63)		10	444	0.76 (0.24, 2.43)		13	902	0.37 (0.15, 0.91)		
aMED				.37				.73				.68				.07	.21
Q1	41	1,984	ref		12	462	ref		5	345	ref		25	846	ref		
Q2	38	1,441	1.16 (0.72, 1.87)		5	359	0.63 (0.14, 2.75)		5	246	0.65 (0.13, 3.19)		16	703	0.80 (0.41, 1.60)		
Q3	74	3,216	1.01 (0.65, 1.58)		18	603	1.57 (0.39, 6.37)		14	661	1.14 (0.28, 4.68)		24	1,117	0.86 (0.42, 1.78)		
Q4	35	1,873	0.84 (0.49, 1.45)		4	309	0.58 (0.08, 4.20)		5	339	0.44 (0.07, 2.66)		7	797	0.24 (0.08, 0.71)		

Abbreviations: CVD (cardiovascular disease), TIA (transient ischemic attack), DQI (diet quality index), Ev (events), Pt (person-time), HR (hazard ratio), CI (confidence interval), tr (trend), int (interaction), Q (quartile), ref (referent), DASH (Dietary Approaches to Stop Hypertension score), hPDI (healthy plant-based index score), HEI (Healthy Eating Index score), ACS (American Cancer Society nutrition guidelines score), aMED (alternate Mediterranean Diet Index score), ER (estrogen receptor), PR (progesterone receptor), HER2 (human epidermal growth factor receptor 2), BC (breast cancer).

[a]Adjusted for age at diagnosis, race/ethnicity, education level, menopausal status, physical activity, smoking status, energy intake, alcohol intake, health plan utilization, comorbidities, cancer stage, ER status, PR status, HER2 status, cardiometabolic conditions, and history of receiving aromatase inhibitors, tamoxifen, and/or radiation therapy.

[b]Participants with any CVD event prior to BC diagnosis were excluded.

[c]There were n=1,427 participants that had no chemotherapy.

[d]From type 3 test using the Wald statistic.

Supplementary Table 3. Subdistribution hazard ratios and 95% confidence intervals for quartiles of baseline diet quality on any CVD event or CVD-related death after breast cancer diagnosis, by radiation status (n=2,906)[a,b]

	Had radiation (n=1,989)				Did not have radiation (n=917)				
DQI	Ev	Pt (yrs)	HR (95% CI)	P_{tr}	Ev	Pt (yrs)	HR (95% CI)	P_{tr}	P_{int}[c]
DASH				.60				.02	.82
Q1	106	4,640	ref		67	2,037	ref		
Q2	147	6,780	0.84 (0.65, 1.09)		64	2,851	0.64 (0.44, 0.94)		
Q3	102	4,755	0.86 (0.64, 1.14)		45	1,836	0.67 (0.44, 1.02)		
Q4	94	5,136	0.76 (0.57, 1.03)		52	2,502	0.62 (0.41, 0.93)		
hPDI				.98				.09	.43
Q1	116	5,250	ref		57	1,995	ref		
Q2	122	6,067	1.02 (0.78, 1.34)		73	2,467	0.99 (0.67, 1.46)		
Q3	116	4,751	1.20 (0.91, 1.58)		47	2,259	0.80 (0.52, 1.22)		
Q4	95	5,243	1.02 (0.76, 1.36)		51	2,506	0.72 (0.47, 1.10)		
HEI				.52				.10	.40
Q1	103	5,136	ref		70	2,126	ref		
Q2	117	5,419	1.00 (0.76, 1.31)		57	2,508	0.77 (0.53, 1.12)		
Q3	114	5,789	0.99 (0.75, 1.31)		51	2,423	0.65 (0.44, 0.95)		
Q4	115	4,968	1.14 (0.86, 1.52)		50	2,170	0.73 (0.49, 1.10)		
ACS				.46				.03	.41
Q1	128	5,466	ref		72	2,225	ref		
Q2	114	5,743	0.89 (0.69, 1.16)		53	2,348	0.57 (0.39, 0.83)		
Q3	109	5,040	0.91 (0.69, 1.19)		54	2,216	0.65 (0.44, 0.95)		
Q4	98	5,062	0.91 (0.69, 1.20)		49	2,439	0.67 (0.46, 0.99)		
aMED				.56				.08	.06
Q1	101	4,749	ref		81	2,066	ref		
Q2	87	3,701	1.11 (0.83, 1.49)		37	1,720	0.60 (0.40, 0.91)		
Q3	169	7,826	1.00 (0.77, 1.32)		71	3,031	0.78 (0.54, 1.13)		
Q4	92	5,035	0.90 (0.65, 1.25)		39	2,410	0.53 (0.33, 0.85)		

Abbreviations: CVD (cardiovascular disease), TIA (transient ischemic attack), DQI (diet quality index), Ev (events), Pt (person-time), HR (hazard ratio), CI (confidence interval), tr (trend), int (interaction), Q (quartile), ref (referent), DASH (Dietary Approaches to Stop Hypertension score), hPDI (healthy plant-based index score), HEI (Healthy Eating Index score), ACS (American Cancer Society nutrition guidelines score), aMED (alternate Mediterranean Diet Index score), ER (estrogen receptor), PR (progesterone receptor), HER2 (human epidermal growth factor receptor 2), BC (breast cancer).

[a]Adjusted for age at diagnosis, race/ethnicity, education level, menopausal status, physical activity, smoking status, energy intake, alcohol intake, health plan utilization, comorbidities, cancer stage, ER status, PR status, HER2 status, cardiometabolic conditions, and history of receiving aromatase inhibitors, tamoxifen, and/or chemotherapy.

[b]Participants with any CVD event prior to BC diagnosis were excluded.

[c]From type 3 test using the Wald statistic.

Supplementary Table 4. Subdistribution hazard ratios and 95% confidence intervals for quartiles of baseline diet quality on any CVD event or CVD-related death after breast cancer diagnosis, by endocrine therapy type (n=2,906)[a,b]

	Any aromatase inhibitors (n=1,850)					Any tamoxifen (n=1,022)					Any endocrine therapy (n=2,227)				
DQI	Ev	Pt (yrs)	HR (95% CI)	P_{tr}	P_{int}[c]	Ev	Pt (yrs)	HR (95% CI)	P_{tr}	P_{int}[c]	Ev	Pt (yrs)	HR (95% CI)	P_{tr}	P_{int}[c]
DASH				.13	.23				.06	.17				.09	.21
Q1	112	4,066	ref			48	2,690	ref			130	5,209	ref		
Q2	155	6,586	0.87 (0.68, 1.13)			58	3,395	0.82 (0.54, 1.24)			166	7,671	0.84 (0.66, 1.06)		
Q3	93	3,962	0.82 (0.61, 1.10)			40	2,430	0.87 (0.56, 1.37)			104	4,955	0.80 (0.61, 1.06)		
Q4	90	4,926	0.70 (0.52, 0.95)			26	2,761	0.53 (0.32, 0.89)			100	6,001	0.69 (0.52, 0.92)		
hPDI				.59	.40				.07	.53				.33	.83
Q1	113	4,690	ref			43	2,678	ref			126	5,689	ref		
Q2	123	5,472	0.95 (0.73, 1.25)			50	3,026	1.03 (0.66, 1.62)			143	6,607	1.02 (0.79, 1.32)		
Q3	112	4,366	1.13 (0.85, 1.49)			46	2,714	1.12 (0.71, 1.76)			123	5,465	1.11 (0.85, 1.45)		
Q4	102	5,012	0.94 (0.70, 1.25)			33	2,857	0.72 (0.44, 1.19)			108	6,074	0.90 (0.68, 1.18)		
HEI				.42	.50				.08	.01				.33	.14
Q1	119	4,343	ref			46	2,632	ref			132	5,515	ref		
Q2	115	5,176	0.86 (0.66, 1.12)			54	3,061	0.93 (0.61, 1.42)			132	6,258	0.90 (0.70, 1.14)		
Q3	110	5,447	0.77 (0.59, 1.02)			44	3,081	0.80 (0.49, 1.30)			122	6,520	0.79 (0.61, 1.02)		
Q4	106	4,574	0.88 (0.67, 1.16)			28	2,503	0.56 (0.33, 0.93)			114	5,542	0.87 (0.66, 1.13)		
ACS				.25	.64				.03	.06				.15	.92
Q1	123	4,861	ref			63	2,967	ref			144	6,004	ref		
Q2	122	5,265	0.86 (0.67, 1.12)			37	2,960	0.51 (0.33, 0.79)			130	6,445	0.81 (0.63, 1.04)		
Q3	113	4,701	0.88 (0.67, 1.15)			40	2,592	0.70 (0.46, 1.07)			124	5,665	0.85 (0.66, 1.11)		
Q4	92	4,712	0.88 (0.66, 1.16)			32	2,756	0.58 (0.37, 0.92)			102	5,721	0.84 (0.64, 1.09)		
aMED				.24	.63				.06	.18				.17	.63
Q1	121	4,212	ref			42	2,247	ref			134	5,101	ref		
Q2	82	3,588	0.86 (0.65, 1.14)			38	2,161	0.87 (0.54, 1.41)			95	4,354	0.91 (0.69, 1.19)		
Q3	166	6,925	0.94 (0.73, 1.22)			68	4,117	0.88 (0.58, 1.35)			180	8,536	0.91 (0.71, 1.17)		
Q4	81	4,814	0.72 (0.52, 1.00)			24	2,751	0.48 (0.28, 0.82)			91	5,844	0.73 (0.53, 0.98)		

Abbreviations: CVD (cardiovascular disease), TIA (transient ischemic attack), DQI (diet quality index), Ev (events), Pt (person-time), HR (hazard ratio), CI (confidence interval), tr (trend), int (interaction), Q (quartile), ref (referent), DASH (Dietary Approaches to Stop Hypertension score), hPDI (healthy plant-based index score), HEI (Healthy Eating Index score), ACS (American Cancer Society nutrition guidelines score), aMED (alternate Mediterranean Diet Index score), ER (estrogen receptor), PR (progesterone receptor), HER2 (human epidermal growth factor receptor 2), BC (breast cancer).

[a]Adjusted for age at diagnosis, race/ethnicity, education level, menopausal status, physical activity, smoking status, energy intake, alcohol intake, health plan utilization, comorbidities, cancer stage, ER status, PR status, HER2 status, cardiometabolic conditions, and history of receiving chemotherapy and/or radiation therapy.

[b]Participants with any CVD event prior to BC diagnosis were excluded.

[c]From type 3 test using the Wald statistic.

Supplementary Table 5. Mutually adjusted subdistribution hazard ratios and 95% confidence intervals for each dietary component on CVD-related death and composite CVD outcomes after breast cancer diagnosis[a,b,c]

	Any CVD event (n=2,906)	CVD-related death (n=3,402)	Any CVD event or CVD-related death (n=2,906)
DQI[d]	HR (95% CI)	HR (95% CI)	HR (95% CI)
DASH			
Low-fat diary	1.00 (0.94, 1.06)	0.85 (0.79, 0.93)	0.98 (0.93, 1.04)
Total fruits	1.01 (0.94, 1.08)	1.00 (0.92, 1.10)	1.00 (0.94, 1.07)
Non-starchy vegetables	0.99 (0.92, 1.06)	0.96 (0.86, 1.07)	1.01 (0.94, 1.08)
Whole grains	0.99 (0.92, 1.05)	1.00 (0.92, 1.10)	1.00 (0.94, 1.06)
Nuts and legumes	0.94 (0.88, 1.00)	0.95 (0.86, 1.05)	0.94 (0.88, 1.00)
Red and processed meats[c]	0.94 (0.88, 1.02)	1.04 (0.94, 1.16)	0.95 (0.88, 1.02)
Sodium[c]	1.06 (0.94, 1.19)	1.01 (0.84, 1.21)	1.04 (0.93, 1.17)
Sweetened beverages[c]	0.98 (0.92, 1.04)	1.00 (0.92, 1.08)	0.98 (0.93, 1.04)
hPDI			
Total diary[c]	0.98 (0.92, 1.04)	1.13 (1.03, 1.23)	0.99 (0.93, 1.05)
Animal fats[c]	1.01 (0.96, 1.07)	0.96 (0.89, 1.04)	1.01 (0.95, 1.07)
Vegetable oils	1.05 (0.99, 1.12)	0.92 (0.85, 1.00)	1.03 (0.97, 1.09)
Fruit juices[c]	0.97 (0.91, 1.03)	0.98 (0.90, 1.06)	0.98 (0.92, 1.04)
Whole fruits	1.00 (0.93, 1.07)	1.01 (0.93, 1.11)	0.99 (0.93, 1.06)
Non-starchy vegetables	0.98 (0.91, 1.05)	0.96 (0.86, 1.06)	1.01 (0.94, 1.08)
Starchy vegetables[c]	0.94 (0.88, 1.01)	0.98 (0.88, 1.09)	0.96 (0.89, 1.02)
Refined grains[c]	1.01 (0.93, 1.09)	1.01 (0.89, 1.13)	1.01 (0.93, 1.09)
Whole grains	0.97 (0.91, 1.04)	0.99 (0.90, 1.08)	0.99 (0.93, 1.05)
Eggs[c]	1.09 (1.02, 1.16)	1.01 (0.93, 1.10)	1.08 (1.02, 1.15)
Legumes	0.93 (0.87, 0.99)	0.96 (0.87, 1.06)	0.94 (0.88, 1.00)
Nuts	0.99 (0.92, 1.06)	0.97 (0.88, 1.07)	0.98 (0.92, 1.04)
Seafood and fish[c]	1.08 (1.01, 1.15)	0.98 (0.89, 1.08)	1.07 (1.00, 1.14)
Total meat[c]	0.91 (0.85, 0.98)	1.01 (0.91, 1.12)	0.92 (0.86, 0.99)
Sweetened beverages[c]	0.99 (0.89, 1.09)	0.95 (0.83, 1.09)	1.00 (0.91, 1.09)
Sweets and desserts[c]	1.00 (0.90, 1.12)	1.09 (0.93, 1.28)	1.00 (0.90, 1.11)
Teas and coffees	1.01 (0.95, 1.08)	1.06 (0.97, 1.16)	1.02 (0.96, 1.08)
HEI			
Total dairy	1.01 (0.98, 1.05)	0.93 (0.88, 0.98)	1.01 (0.97, 1.04)
Saturated fats[c]	1.02 (0.98, 1.07)	0.97 (0.92, 1.03)	1.01 (0.97, 1.06)
Unsaturated fats	0.99 (0.94, 1.03)	1.00 (0.93, 1.06)	0.99 (0.95, 1.03)

Whole fruits	1.00 (0.90, 1.12)	0.95 (0.82, 1.10)	0.99 (0.89, 1.10)
Total fruits	0.96 (0.86, 1.06)	1.05 (0.91, 1.20)	0.98 (0.88, 1.08)
Greens and beans	0.93 (0.84, 1.03)	0.91 (0.80, 1.03)	0.96 (0.87, 1.07)
Total vegetables	1.10 (0.96, 1.26)	1.08 (0.89, 1.30)	1.06 (0.93, 1.21)
Refined grains[c]	0.99 (0.94, 1.03)	1.02 (0.95, 1.10)	0.99 (0.94, 1.03)
Whole grains	0.99 (0.96, 1.02)	1.01 (0.97, 1.05)	1.00 (0.97, 1.03)
Seafood and plant proteins	0.94 (0.85, 1.04)	0.96 (0.84, 1.10)	0.94 (0.85, 1.03)
Total protein	1.01 (0.90, 1.13)	0.96 (0.82, 1.13)	1.02 (0.91, 1.13)
Sodium[c]	1.03 (0.99, 1.07)	1.01 (0.96, 1.06)	1.02 (0.98, 1.06)
Added sugar[c]	1.02 (0.98, 1.07)	1.00 (0.94, 1.08)	1.01 (0.96, 1.06)
ACS			
Total fruits and vegetables	0.92 (0.82, 1.03)	0.83 (0.71, 0.98)	0.92 (0.82, 1.02)
Whole grains	0.97 (0.90, 1.06)	1.01 (0.90, 1.12)	0.99 (0.92, 1.07)
Red and processed meats[c]	0.94 (0.86, 1.02)	0.99 (0.87, 1.11)	0.94 (0.87, 1.03)
SSB + HPF/RG[c]	0.95 (0.85, 1.06)	0.94 (0.81, 1.10)	0.96 (0.86, 1.07)
aMED			
Unsaturated fats	1.04 (0.88, 1.24)	1.04 (0.81, 1.33)	1.02 (0.86, 1.20)
Total fruits	1.04 (0.87, 1.25)	0.99 (0.78, 1.25)	1.01 (0.85, 1.20)
Total vegetables	0.95 (0.79, 1.15)	0.93 (0.73, 1.20)	1.00 (0.83, 1.19)
Whole grains	1.02 (0.86, 1.21)	1.00 (0.78, 1.28)	1.05 (0.89, 1.24)
Legumes	0.89 (0.75, 1.06)	0.87 (0.68, 1.12)	0.90 (0.76, 1.06)
Nuts	0.99 (0.82, 1.18)	0.97 (0.75, 1.26)	0.97 (0.81, 1.15)
Red and processed meats[c]	0.82 (0.68, 0.98)	0.96 (0.74, 1.23)	0.84 (0.71, 1.00)
Seafood and fish	0.86 (0.73, 1.03)	0.92 (0.71, 1.18)	0.86 (0.73, 1.02)

Abbreviations: CVD (cardiovascular disease), DQI (diet quality index), DASH (Dietary Approaches to Stop Hypertension score), hPDI (healthy plant-based index score), HEI (Healthy Eating Index score), ACS (American Cancer Society nutrition guidelines score), aMED (alternate Mediterranean Diet Index score), HR (hazard ratio), CI (confidence interval), SSB (sugar-sweetened beverages), HPF (highly processed foods), RG (refined grains).

[a]All dietary components are mutually adjusted, along with age at diagnosis, race/ethnicity, education level, menopausal status, physical activity, smoking status, energy intake, alcohol intake, health plan utilization, comorbidities, cancer stage, ER status, PR status, HER2 status, and cardiometabolic conditions prior to breast cancer diagnosis.

[b]All subdistribution hazard ratios calculated as a 1-unit change within the dietary quality index component-specific score.

[c]Scores are reversed, whereby higher consumption leads to a lower score.

[d]Specific food components are measured in points, as assigned by each DQI (see Table 1).
